# Supplementary material for: Are carbon emissions trading and green financial instruments synergistic? -Comprehensive quantitative research based on content analysis
Source: PLoS One. 2024 Mar 7;19(3):e0298601. doi: 10.1371/journal.pone.0298601 (PMC10919601; doi:10.1371/journal.pone.0298601)
Supplement: S1 Data — (ZIP) [file pone.0298601.s001.zip › Data/地级市Z_F工作报告.zip!/┴╔─■/╡ñ╢1⁄2╩╨/└┤╫╘╣1⁄2╓┌║┼í╛┬φ┐╦╩2╛▌═°í┐_2019.docx]

各位代表：

　　现在，我代表市政府向大会报告工作，请予审议，并请市政协委员和各位列席人员提出意见和建议。

　　一、2018年工作回顾

　　2018年是本届政府履职施政的开局之年。一年来，我们面对复杂的半岛局势和严峻的经济形势，在省委、省政府和市委的坚强领导下，在市人大、市政协的有力监督下，高举习近平新时代中国特色社会主义思想伟大旗帜，深入学习贯彻党的十九大和十九届二中、三中全会精神及习近平总书记在辽宁考察时和在深入推进东北振兴座谈会上的重要讲话精神，持之以恒落实新发展理念和“四个着力”“三个推进”，深入实施“五大区域发展战略”，坚决落实市第十二次党代会和市委十二届八次全会精神，全力以赴稳增长、促改革、调结构、惠民生、防风险，主要做了以下工作：

　　(一)坚持以“重强抓”统领，全力攻坚克难，经济保持平稳运行

　　面对省政府“重强抓”专项行动这一硬任务，我们坚持“干”字当头、“实”字为先，在省政府下达的120项指标任务基础上，自加压力增加30项任务，一对一跟进、点对点落实，在20多个领域实施“三年行动计划”。改革开放不断深化，乡村振兴战略稳步推进，重振轻工业名城雄风工作成果显现。恒通石墨烯产业园、海洋精密铸造生产线等一批重点项目开工建设，联诚科技高纯氢氧化镁、金汤商业综合体等一批重点项目竣工投产。实现1.2万人脱贫、40个贫困村销号。营商环境建设力度加大，“办事难”“特权车”专项整治成效突出，彰显了政府刀刃向内的决心。大力开展扫黑除恶专项斗争，以创建“百万警民微信群”等措施引领精准打防常态化，万人发案比例全省最低，丹东成为全省最平安的城市之一。荣获“国家卫生城市”称号。

　　面对改革发展稳定中遇到的“硬骨头”，我们坚持迎难而上、破解难题。千方百计筹措资金，想方设法压缩“三公”经费，按时足额发放企业职工养老金。积极协调金融机构和债权人，有效防范丹东港、欣泰电气等企业债务风险，稳定职工就业，为企业重组创造条件。坚决落实中央决策，及时出台调控政策，有效遏制新区“炒房热”。严防死守确保非洲猪瘟疫情“零发生”。坚持“新官理旧账”，市领导包扶攻坚的37个重大历史遗留问题已解决10个，同步开展政府失信和20项民生领域诚信缺失专项治理，促进形成了全社会诚实守信、重信守诺的良好风尚。

　　预计全市完成地区生产总值810亿元，同比增长0.4%;规模以上工业增加值102亿元，增长7%;固定资产投资215亿元，下降10%;社会消费品零售总额601亿元，增长5%;外贸出口总额125亿元，占全省比重3.8%;一般公共预算收入73.4亿元，增长0.9%;一般公共预算支出214.9亿元，增长7.1%;城镇常住居民人均可支配收入29900元，增长7%;农村常住居民人均可支配收入15480元，增长7%。实现了经济持续健康发展和社会大局稳定。

　　(二)坚持创新驱动发展，聚力转型升级，实体经济根基逐步夯实

　　全面实施乡村振兴战略。着眼深化农村集体产权制度等改革，在全省率先构建产权制度管理平台;土地确权面积达332万亩，确权率居全省前列。筹措8000万元支持农村集体经济发展。县域园区集聚效应明显，“飞地经济”发展加快，完善县乡财政管理体制改革，明确三年内新增财力全部返还。着力补齐农村突出短板，新建“四好农村路”110公里，实施农村饮用水安全巩固提升工程和农村环境卫生、“大棚房”综合整治。粮食产量连续5年突破百万吨。大力发展特色高效农业，建设高标准农田9.3万亩，新增设施种植业0.8万亩，无公害、绿色、有机食品生产面积占全市果园和耕地面积的65.6%，农作物耕种收综合机械化水平达到74%，规模以上农产品加工企业达到151家。新增特色经济林593公顷、林下经济253公顷。俄罗斯远洋渔业项目获国家农业农村部批准，渔业经济稳定在全省前列。农产品出口额达到7亿美元，居全省第2位。东港草莓被列入“中国特色农产品优势区”，五龙背镇跻身全国农业产业兴村强镇示范行列，大梨树村入选全国科技示范村。选派479名干部到乡镇和村工作，实施高校毕业生“三支一扶”计划，大力培育新型农民，为乡村振兴提供人才支撑。

　　加快发展现代制造业。狠抓重振轻工业名城雄风工作，推动华泰汽车集团成功并购曙光汽车集团，打造“中国陀飞轮手表制造(丹东)基地”和“中国白醋之乡”2个国家级区域产业品牌，辽宁(丹东)仪器仪表产业基地被评为“测控仪器国家新型工业化产业示范基地”，满族医药研发生产基地建成投入使用。创新驱动成效显著，实施了4个省级重点智能制造及智能服务试点示范项目;省级产业技术创新联盟达到10家、平台达到11个，新增省级企业技术中心2家、工程技术研究中心1家;深化与中科院沈阳分院的合作，20余家企业与13所高校科研院所精准对接，在丹转移转化项目60个。创建国家级高新区工作取得实质性进展。全年新增“小巨人”和“专精特新”企业56户、规模以上工业企业23户;高新技术企业突破120户，增长30%。

　　优化升级现代服务业。旅游业蓬勃发展，打造“山海江田、灵秀丹东”旅游城市名片，凤凰山玻璃索桥、宽甸绿野蓝湾温泉度假村等旅游项目投入运营，全市旅游业总收入增长12%。商贸流通提质增效，网红效应带动安东老街成为“夜经济”最亮点，全市32家重点流通企业夜间消费占比达到36%。线上线下融合发展提速，冷链物流供应链条日益完善，全市电子商务网络交易额增长9%，实物商品网上零售额增长11%，快递业务量增长70%。金融机构本外币贷款余额1323.5亿元。

　　(三)坚持深化改革开放，优化营商环境，发展内生动力不断增强

　　重点改革稳步推进。深入实施供给侧结构性改革，淘汰燃煤锅炉150台，化解房地产库存面积232万平方米，新增电力市场化交易用户111户。完成党政群机构改革和事业单位改革，以及税收征管体制、国有林场等领域改革。在全省率先开展开发区(园区)体制机制改革，建立“小管委会、强开发公司”运营模式。深化国企改革，组建水务集团，努力盘活丹东化纤、丹东纺织等国企资产，启动厂办大集体改革，完成驻丹央企“三供一业”主辅分离移交。

　　对外开放持续扩大。深入开展“开放年”活动，积极参与辽宁沿海六市协同发展，引导企业参与“一带一路”建设，建立海外营销网络16个;水产品、草莓基地晋升为国家级外贸转型升级基地。加快推动《东北东部绿色经济带发展规划》获批，与东北东部地区在港口、物流、旅游等方面开展实质性合作。签署《丹东—扬州对口合作框架协议》。《国家物流枢纽和建设规划》将丹东列为陆上边境口岸型国家物流枢纽城市。

　　招商引资成效明显。开展市级层面“走出去，请进来”招商活动50余次，围绕旅游资源开发、智慧城市、港口物流、企业重组等方面，精准包装推介项目300余个，与韩国SK集团、中交建集团、东软集团、中高集团、中国燃气、西电集团等知名企业对接洽谈，虎山长城相遇文化小镇等31个亿元以上大项目签约落地，与中国燃气确定了重组市燃气公司方案，与东软集团合资成立了智慧城市运营公司。全年引进内资到位资金219亿元。

　　营商环境不断优化。深入推进“放管服”改革，大力清理行政职权，全力以赴助力实体经济发展，出台加快民营经济发展24条新政，落实各项税费减免约20亿元。实施“项目管家”制度，推行“五个一”工程、“四联合三多一简化”审批，实行审批窗口双休日无休服务，推动简化流程与提高效率并举，践行了“只进一扇门”“最多跑一次”的诺言。整合民心网、12345市民热线，8890政务便民服务平台正式开通，解决企业和群众痛点问题近200个。深化商事制度改革，实现“32证合一”，全面推行“证照分离”改革，完成“个转企”813户，近百个银行网点实现工商登记全程电子化一站式办理，新增各类市场主体4.3万户。

　　(四)坚持以人民为中心，厚植民生沃土，百姓幸福指数明显提升

　　社会保障体系臻于完善。城镇新增就业1.7万人，城镇登记失业率4.66%，零就业家庭保持动态为零。养老金“十四连涨”、城乡低保“十三连涨”，抚恤优待等救助标准稳步提高。发放残疾人“两项补贴”3000余万元，为2万余名残疾人免费办理意外伤害保险。“丹东惠民卡”APP上线运行，远程认证率达到78.3%。养老服务业发展加快。完成棚户区住房改造1647套、农村危房改造2140户、老旧小区改造17个。

　　城市建设管理日益精细。完成锦山大街、黄海路等道路提升改造，宝山大街、江城大街等9处低洼易涝区改造，桃源立交桥、东尧桥等10座桥梁维修改造，以及四号干线续建主体工程。完成主要街路及小区供水管网改造22公里;全线贯通应急输水管线，实现第二水源向市区水厂供水。完成丹东客运站维修改造，建设公交停车场3个、公共停车位1000个。完成裸土覆绿、山体生态修复工程2处，新增绿地面积1万平方米;建成新区鸭绿江彩色健身步道4.5公里。持续开展占用非机动车道、小广告、露天烧烤等专项整治，拆除广告牌匾450处;加大交通综合整治力度，向道路拥堵等“城市病”开刀。

　　社会事业全面进步。全市星级幼儿园达到289所，改造农村义务教育薄弱学校58所，国家级义务教育发展基本均衡县(市)区全部通过验收，辽宁机电职业技术学院入选全国首批“行业骨干职业院校”。启动“健康城市”建设，持续扩大新农合异地就医即时结算覆盖面，建成标准化乡镇卫生院27家，18家医保定点医疗机构与国内8600家相应机构联网直接结算，城市和县域医联体分别达到4个、7个，实现政府办基层医疗卫生机构全覆盖。建成乡镇(街道)、村(社区)综合性文化示范点30个，放映农村电影8000余场(次)，建设小型专业博物馆10个。全民健身活动日益活跃，配备健身路径50套，启动示范性体育公园建设。持续创建国家食品安全示范城市。安全生产、信访形势总体稳定。国防和民兵预备役工作取得新成绩。

　　(五)坚持绿色发展理念，突出生态建设，环境质量位居全省前列

　　牢固树立“绿水青山就是金山银山”的理念，加强生态环境保护。完成生态红线划定，红线比例为50.8%。中央环保督察和国家海洋督察反馈问题按时间节点完成整改;“绿盾2018”自然保护区违法违规问题整改取得实效。开展鸭绿江流域环境整治，坚决关停污染严重的丹东化纤;建立水污染企业黄红牌公示制度;完成花园河、五道河、白房河建成区段清淤及凤城、宽甸污水处理厂提标改造。全面落实“河长制”，流域面积10平方公里以上的435条河流全部设立河长。10个县级以上集中式饮用水水源地水质达标率100%。城区优良天数率95.8%，环境空气质量居全省首位。城市垃圾无害化处理率100%。刘家河镇被评为第一批省级特色乡镇，毛甸子镇、大西岔镇、通远堡镇被评为省级生态乡镇;窑沟村被评为中国美丽休闲乡村，宏天村等25个村被评为省级生态村，绿色发展呈现可喜局面。

　　(六)坚持思想政治引领，强化责任担当，政府自身建设持续加强

　　认真学习贯彻落实党的十九大精神及习近平总书记在辽宁考察时和在深入推进东北振兴座谈会上的重要讲话精神，出台政府系统贯彻落实总书记重要讲话精神系列方案。深入开展“解放思想 推动高质量发展大讨论”。全力以赴做好中央巡视反馈意见整改工作。扎实推进“两学一做”学习教育常态化制度化，全面落实政府系统党风廉政建设主体责任，政府系统广大党员干部 “四个意识”更加牢固、“四个自信”更加坚定、“两个维护”更加自觉。严格落实政府系统意识形态工作责任制。坚持依法科学民主决策，全面落实重大决策合法性审查和“三重一大”制度。自觉接受市人大及其常委会的依法监督和市政协的民主监督，主动听取民主党派、工商联、无党派人士和人民团体的意见建议，622件人大代表建议和政协提案全部办结。推进审计监督全覆盖。政务公开不断深化。应急管理全面加强。社会矛盾纠纷排查化解率达到99%。政府法治建设、效能建设、诚信建设、廉政建设迈上新台阶，政府凝聚力、公信力、执行力、战斗力得到全面提升。

　　这些成绩的取得，得益于省委、省政府和市委的坚强领导，得益于市人大、市政协的监督支持，得益于全市人民的共同努力。在此，我代表市政府，向全市人民，向人大代表、政协委员，向各民主党派、工商联、无党派人士和各人民团体，向中省直部门、解放军驻丹部队，表示衷心的感谢并致以崇高的敬意!

　　在总结成绩的同时，我们也清醒地认识到，当前丹东正处于经济转型升级、爬坡过坎的关键时期，我们的总体发展水平与高质量发展要求相比还有不小的差距，对外开放水平与沿海兄弟城市相比还有不小的差距，基本公共服务水平与群众要求相比还有不小的差距。突出表现在：一是半岛形势复杂多变对我市经济冲击严重，尤其是外贸进出口大幅下滑，开拓新市场尚待时日;二是招商引资和项目建设受外部环境影响，洽谈的多、落地的少，年初确定的开复工项目没达到进度要求，项目支撑发展后劲不足;三是财政增收空间较小，养老金支出、政府性债务负担较重，收支矛盾依然突出;四是医疗、教育、交通、住房等民生领域还存在短板;五是部分干部干事创业热情不足，不想为、不敢为、不作为、慢作为现象和违纪违法案件时有发生。这些问题已引起我们的高度重视，将采取有力措施加以解决。

　　二、2019年全市经济社会发展目标任务和重点工作

　　各位代表，2019年是新中国成立70周年，是贯彻落实党的十九大精神的深化之年，是决胜全面建成小康社会的攻坚之年，也是推进新时代丹东振兴发展的关键之年。今年政府工作的总体思路是：坚定不移贯彻落实习近平新时代中国特色社会主义思想，特别是习近平总书记关于东北、辽宁振兴发展一系列重要指示批示精神，统筹推进“五位一体”总体布局，协调推进“四个全面”战略布局，坚持稳中求进工作总基调，坚持新发展理念，坚持推动高质量发展，坚持以供给侧结构性改革为主线，坚持深化市场化改革、扩大高水平开放, 按照市委十二届十二次全会的部署要求，解放思想、锐意进取，深入开展“重强抓”专项行动，进一步稳就业、稳金融、稳外贸、稳外资、稳投资、稳预期，保持经济运行在合理区间，着力提高人民群众获得感、幸福感和安全感，以新气象新担当新作为推进丹东全面振兴、全方位振兴。

　　今年经济社会发展的主要预期目标是：地区生产总值增长与全省同步，规模以上工业增加值增长8%，固定资产投资增长8%，社会消费品零售总额增长7%，外贸出口总额占全省份额不减，一般公共预算收入增长6.5%，城镇常住居民人均可支配收入增长7%，农村常住居民人均可支配收入增长7%。

　　为了完成上述目标任务，我们将全力抓好以下工作：

　　(一)强化政策对接，抢抓发展新机遇

　　——高起点站位。以习近平总书记在辽宁考察时和在深入推进东北振兴座谈会上的重要讲话，及国家新一轮支持东北老工业基地振兴为契机，以“解放思想 推动高质量发展大讨论”成果转换为动力，以半岛形势回暖为机遇，在拉长补齐 “四个短板”和落实“六项重点工作”上求实效，谋划和引领丹东的全面发展和全面振兴。

　　把握发展大势，抢抓发展机遇。重点对国家、省各项利好丹东政策进行再梳理、再挖掘、再推进、再落实。加快对接《国家物流枢纽和建设规划》政策，尽快将丹东建设成为一流的陆上边境口岸型国家物流枢纽城市;全力做好《东北东部绿色经济带发展规划》《辽宁丹东重点开发开放试验区建设实施方案》申建及承接工作;超前谋划《辽宁“一带一路”综合试验区建设总体方案》中涉及丹东的7件事;深入实施兴边富民行动，推进边民生产生活改善、基本公共服务提升、重大基础设施建设和优势产业培育等系列工程，推动边境民族地区加快发展。全面激活沿江沿海沿边优势、资源禀赋优势、产业基础优势和交通设施优势，切实把上级政策优势和丹东自身优势转化为经济发展优势。

　　(二)加快改革攻坚，优化营商新环境

　　——高效能工作。巩固和扩大机构改革、事业单位改革成果，加快转变政府职能，创新事中事后监管，继续推进体制机制改革，破除影响效能提升的痛点堵点。落实好《丹东市党政干部容错纠错暂行办法》，促进政府系统干部人岗相适、人事相宜。深化“互联网+政务服务”，让企业和群众办事像“网购”一样方便，全力打造省内一流营商环境。

　　在全面深化改革中释放活力。坚持以供给侧结构性改革为主线不动摇，运用市场化、法治化手段，在“巩固、增强、提升、畅通”上下功夫。推动产能过剩行业加快出清。继续推进国有企业改革，完善市属企业集团现代企业管理模式，加大力度解决国有企业历史遗留问题，启动市燃气总公司改制，持续推进厂办大集体改革。切实增强微观主体活力，促进正向激励和优胜劣汰。继续深化财税、农业农村、科技体制等改革。全力促进市场和生产主体、经济增长和扩大就业、金融和实体经济良性循环，为发展活力大释放提供原动力。

　　在优化营商环境中激发潜力。积极复制辽宁自贸试验区第一批改革创新经验做法。落实更大规模的减税降费政策，最大限度降低营商成本。持续深化“放管服”和商事制度改革，建立“企业+项目管家+相关部门”的服务模式。全面推行审批流程标准化，推进“一网、一门、一次”改革，市县级政务服务事项网上办理率达到80%以上、政务服务事项“一窗”分类受理率达到70%以上、100个高频审批事项“最多跑一次”，打通服务的“最后一公里”。

　　(三)壮大实体经济，培育产业新动能

　　——高质量发展。坚持“两个毫不动摇”，以创新引领经济发展和产业升级，加大科技支撑和投入，全力做精一产、做强二产、做优三产，积极推动优质资源向实体经济集聚、政策举措向实体经济倾斜、工作力量向实体经济强化, 培育新的经济增长点，加快形成多极支撑的现代化产业体系。

　　坚持品牌驱动，做精现代农业。扎实推进乡村振兴战略，坚持农业农村优先发展。调好调顺调优农业结构，推动农业生产由增产转向提质。培育一批以小浆果、中药材、食用菌为主的特色产业集群和特色农产品生产基地，集中力量将草莓、蓝莓、软枣猕猴桃等小浆果做成辽宁乃至全国最大的优质生产基地和种苗繁育基地;建设一批以辽宁绒山羊、沿江牛、辽丹黑猪、中华蜜蜂为主的 “育、繁、推”一体化发展示范场;打造一批连片美、持久美的丹东新农村，新增省级美丽乡村示范村15个。全面推广健康生态养殖，加快浅海滩涂增养殖、海水池塘立体养殖等特色渔业基地建设，探索打造丹东渔港经济区。提升农产品加工和出口水平，农产品加工集聚区主营业务收入增长7%以上。加强“菜篮子”工程建设，加快推进无公害、绿色、有机农产品和地理标志农产品认证，重点借助“舌尖上的丹东”等媒介平台栏目大力宣传优质特色农产品，做大做强丹东农业区域公用品牌，全面叫响丹东大米、梭子蟹、大黄蚬、草莓、板栗、石柱参等优质品牌。实施农村科技特派行动，普及中国农技推广APP，建设农业科技示范服务平台。切实将农业的潜在优势转化为现实的经济优势。

　　坚持创新驱动，做强现代制造业。盯住“重振轻工业名城雄风”目标不放松，聚焦有基础、有优势的轻工业，坚持做大总量与提高质量并重、发挥优势与补齐短板齐抓、培育新兴产业与改造传统产业共管。壮大产业集群规模，省级产业集群增加值增速超过全省平均水平。打好“小升规、规升巨、强龙头、育集群”组合拳，新增“小升规”企业20户以上、“小巨人”和“专精特新”企业50户以上。着力提升“老字号”、开发“原字号”、壮大“新字号”，实施更多的智能制造及智能服务试点示范项目，推动更多的企业达到工业4.0标准，打造更多的行业区域品牌和企业品牌。以创建国家级高新区为目标，积极引导高新技术产业和战略性新兴产业向高新区集聚，年内高新技术企业达到135户，科技进步对经济增长的贡献率达到56.5%，加快实现高新技术产业从“模仿者”“追赶者”到“并行者”“领跑者”的转变。

　　坚持多轮驱动，做优现代服务业。深入实施服务业发展三年行动计划，推动生产性服务业向专业化高端化提升、生活性服务业向智慧化品质化转变、公共服务业向规范化便利化发展。突出旅游业龙头引领作用，打好“旅游牌”，做足“旅游+”“+旅游”的大文章，把更多的历史文化元素融入旅游开发，强化景区景点管理，把“点”做出精品、把“线”做出品牌、把“面”做成特色，真正打造一批精品景区、精品线路、精品套餐;重点推进天桥沟创建国家5A级旅游景区，加快蒲石河森林公园、刘家河温泉水镇、北黄海度假村二期、宽甸全域旅游综合服务区等重点旅游项目建设，积极申创国家边境旅游试验区，加快凤城、宽甸“国家全域旅游示范区”创建，举办好鸭绿江国际旅游节、河口桃花节、天桥沟枫叶节、东港观鸟节等精品节庆活动，在全国叫响“山海江田、灵秀丹东”的旅游城市品牌。大力发展电子商务，推动凤城、宽甸申创国家电子商务进农村综合示范县，推进国门湾互贸区跨境电子商务产业基地和东港黄海水产品批发市场跨境海鲜电商基地建设，复制大连跨境电商综合试验区建设经验，设立丹东电子商务口岸国际贸易“单一窗口”，建设 “丹东跨境电子商务通关服务平台”。持续依托生态资源、温泉森林资源，积极引导社会力量参与养老服务，推进医养结合，加快健康养老服务产业发展。

　　坚持政策驱动，壮大民营经济。大力支持本土民营企业发展，着力在破解制约民营企业发展的痛点、堵点、难点问题上下功夫，落实好“优化环境、降低成本、金融支持、创新转型、保护合法权益、政策保障”6个方面的扶持民营企业政策，坚决兑现“一般性经营企业开办时间压缩至3个工作日、有效降低企业用工成本”等政策。进一步清理规范涉企收费，降低民营企业用工、用能、用地、物流成本。打破各种“卷帘门”“玻璃门”“旋转门”，放宽市场准入限制，实现“非禁即入”。引导金融机构加大对民营企业信贷投放力度，切实解决民营企业和小微企业融资难融资贵问题，全市实现各类融资60亿元。大力营造依法保护企业家合法权益的法治环境、促进企业家公平竞争诚信经营的市场环境、尊重和激励企业家干事创业的社会氛围。

　　(四)实施开放引领，开创招商新局面

　　——高层次开放。牢牢把握开放这一丹东最大的优势和资源，在共建“一带一路”和东北亚开放的大背景下，全方位谋划对外开放，着力打好“开放牌”，做足“边”的文章，释放“海”的潜力，激发“江”的活力，以全面开放引领全面振兴。

　　着力在全面开放上见成效。坚持与沿海兄弟市协同发展，积极参与辽宁“一带一路”综合试验区、中国—中东欧“16+1”经贸合作示范区建设。突出港口在开放中的拉动作用，以辽宁港口整合为契机，加快丹东港重组步伐。组织企业“走出去”参加国内外出口商品展销会，重点开拓东盟、中东欧、非洲和南美进出口市场，支持汽车零部件、医药化工、纺织服装、食品加工等行业企业到中东欧、东南亚和非洲投资，推进安德食品、永明食品、时代龙增压器等企业在日韩、美国、西班牙、阿联酋等国家开展海外仓储业务。加快丹东新区发展，推动对外开放平台建设和重点口岸升级，完善国门湾中朝边民互市贸易区基础设施，加快申建综合保税区，着力将丹东打造成对朝鲜半岛开放的前沿先导区。

　　着力在深化合作上下功夫。强化与东北东部区域城市合作，积极对接吉林“向南发展”战略，加快丹东港在东北东部的陆港建设，推进“白通丹”区域经济一体化发展、东北东部区域物流一体化进程和鸭绿江旅游联盟建设。主动对标先进，近学大连、远学扬州，学习其先进理念、创新思维、体制机制，借鉴其在对外开放、营商环境建设、国企和民营经济发展等方面的经验做法，直接复制推广一批行之有效的改革创新举措。

　　着力在招商引资上见真章。坚持把招商引资作为“一号工程”，瞄准国家重大战略方向、产业导向和宏观政策走向，深度包装200个重点项目;盯住长三角、珠三角、京津冀等发达地区，重点推进智慧城市、中韩SK产业园、孔雀谷时间小镇、华润大东沟水体治理、水下大数据中心等一批10亿元以上大项目尽快落地，全年引进内资到位资金240亿元。在引进外资上，探索建立海外企业库和招商代理人制度，适时设立驻日韩项目联络处。实施重大人才工程，创新人才评价激励机制，放宽事业单位选人用人自主权，加强“丹东工匠”队伍建设，积极引导在外丹东籍企业家返乡发展。坚持把园区作为招商的重要平台，错位布局园区功能，引导项目向园区集聚。积极发展“飞地经济”，重点做好顶层设计及规划、利益分配、考核等相关配套政策的制定;县域全部建立“飞地园区”，80%的乡镇年内有“飞地项目”落地;以更宽的视野探索在港口附近发展省际“飞地经济”，依托东北东部腹地资源和丹东港出海大通道优势，主动承接京津冀、江浙沪等发达地区产业转移。

　　着力在项目建设上求突破。牢固树立“项目至上”理念，坚持储备项目抓前期、招商项目抓落地、新建项目抓开工、续建项目抓进度。年内实施5000万元以上项目270个，计划投资总额1113亿元，其中新开工项目115个、计划投资总额310亿元。重点抓好丹通高铁、本宽高速、沿江公路贯通、丹前铁路外移、鸭绿江水源地上移、丹东水源引入东港等项目的推进工作;润恒冷链物流、虎山长城相遇文化小镇、高新烯碳材料、凤东线升级改造、安东老街大街区等项目的开工建设;丹东金山湾至本溪段既有铁路改造、抗美援朝纪念馆改扩建、康辰药业中药产业园、好食鸡食品深加工等项目竣工投产。切实抓好项目服务，把领导联系重点项目、“项目管家”、项目调度等制度落实到位、执行到位。

　　(五)严守生态底线，建设人居新佳地

　　——高规格保护。把生态环境作为丹东最大的后发优势，牢牢抓住绿色发展这个根本点，坚决打好污染防治攻坚战，推动生态文明建设继续走在全省前列。

　　坚决抓好督察反馈问题整改。以问题为导向，深入落实生态环境保护责任，高标准、高质量按期完成中央环保督察、国家海洋督察反馈问题和生态环保督察“回头看”问题整改落实。继续保持近岸海域水质优良比例不下降;巩固市级水源地整治成果，完成马市岛污水收集处置和县级水源地污染整治销号。

　　坚决打好蓝天、碧水、青山、净土、农业农村污染治理攻坚战。继续淘汰县级建成区20吨及以下燃煤小锅炉，加强机动车污染防控。加快推进大洋河等重点流域水利工程建设;启动市污水处理厂(二期)工程，孤山和新区污水处理厂投入运营;持续开展鸭绿江口水环境综合整治，保护好鸭绿江水源地，全市河流水质优良比例保持在87.5%、城市集中式饮用水水源地水质优良比例保持在100%、城市内河黑臭水体控制在10%以内。实施森林质量精准提升工程和乡村绿化美化工程。深入开展非煤矿山整治。学习借鉴浙江“千万工程”经验，实施村庄清洁行动，抓好“三清一改”，完成农村环境治理项目207个，保护好农业农村资源。

　　(六)补齐民生短板，共享振兴新成果

　　——高水平保障。着力解决人民群众最盼最急最忧最怨的问题，推动财力向民生集中、政策向民生倾斜、服务向民生覆盖。

　　持续巩固脱贫攻坚成果。落实贫困人口和贫困村扶持政策措施，开展建档立卡贫困人口脱贫质量普查，有效减少和防止贫困人口返贫，确保国家现行标准下农村建档立卡贫困人口“两不愁三保障”。

　　稳步提高社会保障水平。把稳就业摆在突出位置，优化公共就业服务，推进创业带动就业，城镇新增就业1.7万人，城镇登记失业率控制在5.2%以内。继续做好农民工工资支付保障工作。企业养老保险参保覆盖99万人。持续提高城乡低保、社会救助、抚恤优待标准;开展残疾人精准康复服务行动。坚持房住不炒，建立购租并举的住房制度，新开工棚改房222套，改造老旧小区15个、农村危房1526户。切实提高住宅小区物业管理水平。

　　全面提升城乡功能品质。不断拉长补齐城市基础设施短板，继续开展地下管网、给排水、供热等城市公用设施建设改造，启动市垃圾处理场改造(焚烧发电项目)及东港、凤城垃圾焚烧项目，开工建设宽甸县热电联产项目。优化城乡路网结构，实现四号干线通车，升级改造滨江路、桃源街等城市道路20条，加固提标民主桥、七道桥等城市桥梁，启动干线公路与铁路“平改立”项目5处，新建“四好农村路”100公里。继续解决城市行车难、停车难问题，加快电动车充电桩建设，新建城市停车位800个，推进东泰路、东尧桥、新区公交枢纽站建设。做好断桥至五道河段鸭绿江景观带提升改造和亮化工程，实施城市内河护岸整治及绿化美化工程。持续提升城市精细化建设管理水平，加快智慧城市和城市管理数字化平台建设。严控老城区及沿江一线高层建筑，保护好有文化价值的历史建筑。巩固国家园林城、国家卫生城创建成果，持续创建全国文明城和环保模范城。

　　大力发展社会事业。推进教育现代化，科学规划城区教育布局，义务教育学校标准化建设动态监测达标率达到95%，实施实验小学、第十九中学扩建和新区初中建设;实现乡镇公办中心幼儿园全覆盖，普惠性幼儿园学位覆盖率达到77%;基本完成“全面改薄”任务;加强名师队伍建设，铺开中小学课后服务;推进职业教育办学体制改革。完善城乡公共卫生服务体系，完成全市乡镇卫生院标准化建设任务，继续推进基层疾控机构、妇幼保健机构和中医馆标准化建设;启动市妇女儿童医院异地新建、市中心医院急救中心建设;引进培养全科医生100名，城乡每万名居民拥有3名全科医生。大力弘扬社会主义核心价值观，丰富人民群众文化体育生活，拓展文化惠民工程，启动市图书馆、市档案馆建设;继续开展全民健身活动。加强国防安全教育，统筹推进国防动员，继续推动军民融合发展，切实做好双拥和退役军人管理保障工作。

　　(七)化解重大风险，构建防控新体系

　　——高标准防控。把维护边境地区安全稳定作为最大的政治任务，强化红线意识，坚守底线思维，主动解决源头性、基础性问题，确保社会长治久安、人民安居乐业。

　　严密防范财政金融风险。严格实行政府债务预算管理和限额管理，守住举债红线，有效降低财政隐性债务风险。千方百计筹措资金，弥补养老金缺口，确保养老金按时足额发放。强化金融秩序监管，坚决打击非法集资、金融诈骗等违法犯罪行为，做好重点企业债务风险防范工作，坚决守住不发生系统性金融风险的底线。

　　严格防范社会风险。全面加强应急处突能力建设。强化重点行业领域安全专项治理，坚决遏制重特大生产安全事故发生。进一步加强食品生产、流通、餐饮各环节监管，持续创建国家食品安全示范城市。切实加强边海防管控。深入开展扫黑除恶专项斗争，实现转递黑恶线索核查率100%。

　　三、注重担当实干，建设人民满意的法治政府

　　加强政府自身建设，需要我们不忘初心、牢记使命，敢于刀刃向内、自我革命，以“功成不必在我”的境界，多做打基础、利长远的事情，努力创造经得起实践、人民、历史检验的实绩。

　　坚持讲政治，强化思想引领。自觉以习近平新时代中国特色社会主义思想为引领，增强“四个意识”，坚定“四个自信”，践行“两个维护”。严守政治纪律和政治规矩，全面加强政府系统意识形态工作，牢牢把握正确的政治方向。坚持解放思想、开拓创新，以思想上的“破冰”推动行动上的“突围”，着力形成一批推动发展的“金点子”和解决问题的“好招数”，以等不起的责任感、慢不得的紧迫感、坐不住的危机感、误不起的使命感，全力推动上级决策部署在丹东落地生根、开花结果。

　　坚持讲法治，深化依法行政。将政府工作全面纳入法治轨道，不断提高运用法治思维和法治方式推动工作的能力。认真执行市人大及其常委会的决议决定，自觉接受市人大和政协监督，广泛听取各民主党派、工商联、无党派人士和人民团体意见和建议，主动接受司法、社会和舆论监督。全面落实政府法律顾问制度，做好政府立法和应诉工作，加强行政复议规范化建设，规范行政执法行为。健全政务公开制度，畅通政府和公众互动渠道，全面提高政府公信力。

　　坚持讲担当，持续改进作风。带头发扬雷厉风行、马上就办的良好作风，持续深化“重强抓”专项行动，用好督查“利器”和考核“指挥棒”，确保政府工作快捷高效、落实有力。大力弘扬大梨树“干”字精神，引导政府干部重实干、出实招、求实效，只为能行想办法，不为不行找借口，树立起凝神聚力谋发展、全神贯注搞建设的鲜明导向。坚决贯彻科学精细、追求卓越的工作理念，激发工作激情，提振干部士气，提高工作水平，努力形成比学赶帮、奋力争先的良好氛围。积极倡导勇于负责、敢于攻坚的担当精神，继续开展历史遗留问题攻坚、失信突出问题和不担当不作为问题专项治理，落实正向激励和容错纠错机制，旗帜鲜明地为干事者担当、为作为者撑腰，推动政府作风持续优化。

　　坚持讲廉洁，严守从政底线。严格落实全面从严治党主体责任，推动政府系统党风廉政建设向纵深发展。做好巡视反馈意见的持续整改。严格执行中央八项规定精神，严控“三公”经费和一般性支出。实现审计监督全覆盖，严防重点领域腐败问题。全面净化政府系统政治生态，营造风清气正、昂扬向上的干事创业氛围。

　　各位代表，新思想引领新时代，新作为谱写新篇章。我们要高举习近平新时代中国特色社会主义思想伟大旗帜，认真贯彻落实习近平总书记在辽宁考察时和在深入推进东北振兴座谈会上的重要讲话精神，在市委的坚强领导下，勠力同心、真抓实干，攻坚克难、奋力赶超，以优异的工作业绩迎接中华人民共和国成立70周年!
